# Exploring Feature Priorities and User Needs in Developing Virtual Study Assistants

**DOI:** 10.2196/86945

**Published:** 2026-03-06

**Authors:** Chi-shan Tsai, HyunHae Lee, Warren Szewczyk, Julia K Palmer, Sophie Putnam, Sean A Munson, Jaimee L Heffner, Alexi Vasbinder, Amandalynne Paullada, Weichao Yuwen, Kerryn W Reding

**Affiliations:** 1 School of Nursing University of Washington Seattle, WA United States; 2 Department of Microbiology University of Washington Seattle, WA United States; 3 Department of Human Centered Design & Engineering University of Washington Seattle, WA United States; 4 Public Health Sciences Division Fred Hutchinson Cancer Center Seattle, WA United States; 5 Department of Linguistics University of Washington Seattle, WA United States; 6 School of Nursing & Healthcare Leadership University of Washington Tacoma Seattle, WA United States

**Keywords:** virtual study assistant, user preference, user need, user-centered design, generative artificial intelligence

## Abstract

This formative research explored health science researchers’ perspectives on the development of an artificial intelligence–based virtual study assistant and identified 8 potential features and their priorities.

## Introduction

With the proliferation of generative artificial intelligence (GenAI) tools in recent years, many research teams are exploring their potential applications in the health care field, including medical education, information provision, and disease diagnosis [1,2]. For example, GenAI tools have been used to support health care research training [3]. One underexplored application of GenAI is an AI-based virtual study assistant (VSA), which we define as a conversational and agentic technology capable of supporting participant-facing tasks in clinical research, such as screening, providing information, and facilitating consent. The purposes of this formative research were to (1) explore health science researchers’ perspectives on the development of an AI-based VSA and (2) identify potential features and their priorities for future AI-based VSA prototype development.

## Methods

### Study Design

Participants were recruited from the University of Washington (UW) and consisted of research investigators and study staff with at least 2 years of experience in human subjects research. A snowball approach was used for recruitment. The individuals first completed a questionnaire (Multimedia Appendix 1) collecting information on their research experience and prior experience with GenAI. One-hour focus groups were conducted to identify a list of potential features for AI-based VSA guided by semistructured questions (Multimedia Appendix 2) about key features and participants’ perceptions related to their use. In the final stage, participants completed follow-up surveys (Multimedia Appendix 3) to assess feature acceptability and preference across studies of varying risk levels.

Quantitative data were analyzed using Microsoft Excel. Descriptive statistics, including frequency and mean (SD), were calculated. Thematic analysis was used to identify potential features in the qualitative interview data [4]. Two researchers coded the transcripts and identified themes. The prioritization of preferred features was evaluated using Borda Count, a ranking-based scoring method.

### Ethical Considerations

This study was deemed exempt by the University of Washington Institutional Review Board (#21197) since it involved only minimal risk interviews and surveys. Informed consent was obtained from all participants, and data were recorded without identifiable information. All participants received a $10 gift card for completing the survey and a $25 gift card for participating in the focus group.

## Results

A total of 14 respondents completed the pre–focus group survey. Among them, 10 took part in focus groups (n=5 per group). The others could not attend due to scheduling conflicts. Following the focus groups, a follow-up survey was distributed to all pre–focus group survey respondents, and 11 completed it. Focus group participants mostly included faculty members (n=6) or student research assistants (n=3). Participant demographics and work experience are presented in Table 1.

**Table 1:** Demographics and research experience by study activity (with overlapping participants).

Characteristics		Pre–focus group survey (n=14), n	Focus group (n=10), n	Follow-up survey (n=11), n
**Gender**				
	Female	11	8	9
	Male	2	1	1
	Nonbinary	1	1	1
**Race**				
	Asian	2	1	2
	Black or African American	1	1	0
	White	9	7	7
	More than one racial group	1	1	1
	Prefer not to answer	1	0	1
**Ethnicity**				
	Hispanic or Latino	1	0	1
	Not Hispanic or Latino	13	10	10
**Job title**				
	Research scientist/principal investigator	8	5	5
	Interventionist	1	1	1
	Research coordinator/assistant/consultant	4	3	4
	Other	1	1	1
**Education**				
	Bachelor's degree or other 4-year college degree	1	1	1
	Master’s degree	6	5	6
	Doctoral degree	7	4	4
**Work experience**				
	2-3 years	3	2	3
	5-6 years	1	1	1
	6-7 years	1	1	0
	7-8 years	2	1	2
	9-10 years	3	1	3
	10 years	4	4	2
**Types of human subjects studies^a^**				
	Interventional studies such as clinical trials	9	6	8
	Dissemination and implementation trials	5	4	4
	Observational studies with biospecimens or behavioral testing	7	6	5
	Observational studies with surveys only	9	5	7
	Qualitative studies	12	8	10
	Secondary data analyses, electronic medical record studies, or similar	4	3	2
**Remote study experience**				
	Yes	10	7	8
	No	4	3	3
**Risk category experience^a^**				
	More than minimal risk (full institutional review board review)	—^b^	—	5
	No more than minimal risk and exempt	—	—	9

^a^Multiple responses were allowed.

^b^Not applicable.

**Figure 1:**
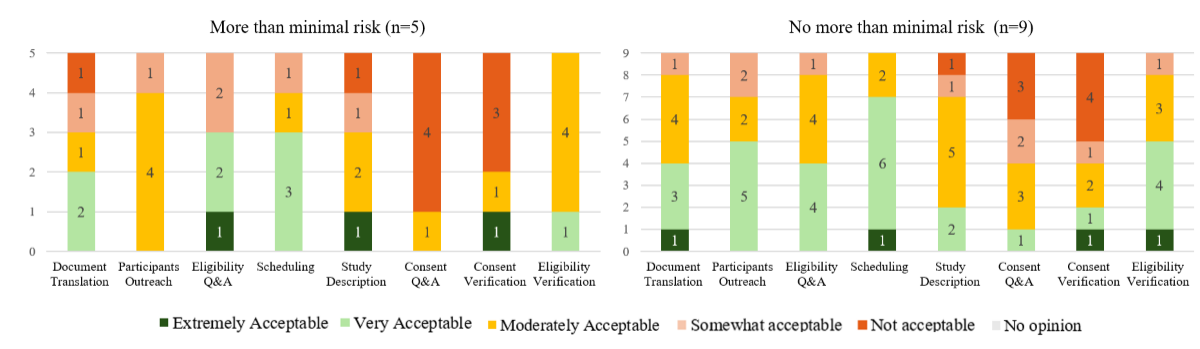
Acceptability of artificial intelligence–based virtual study assistant features among participants completing follow-up survey, by risk level of human subjects research. Q&A: questions and answers.

Eight potential features for the AI-based VSA were identified from focus group responses, presented in order of most acceptable to least: (1) translating study documents into other languages, (2) contacting a potential participant to gauge their interest, (3) asking and answering questions about eligibility for the study, (4) scheduling participant interactions, (5) describing the study to participants, (6) answering participant questions about the consent form or study participation, (7) verifying participant understanding of consent, and (8) verifying eligibility. Most features were generally considered acceptable. However, those related to answering participant questions about the consent form or study participation, as well as consent verification, received lower levels of acceptance, particularly in studies involving more than minimal risk (Figure 1).

## Discussion

Our findings reveal promising directions for the development of AI-based VSA for human subjects research. Among the potential features, translating study documents into other languages was rated as the most preferred. Features related to participant outreach and eligibility-related questions also ranked relatively high, indicating preferences for tools that reduce administrative burden and improve participant communication. One previous study [5] explored the use of GenAI for translating medical or public health–related documents in the health care sector. Specifically, postediting machine translation was on average 14% faster than translating from the beginning. Only a small proportion of outputs (11%-16%) required no human edits. Approximately half of the words needed to be edited [5]. These findings revealed both the potential and the limitations of such tools in this domain. Future research regarding VSA development could explore the linguistic and cultural considerations. There is limited research on using GenAI for participant outreach and scheduling; however, if such features were to be designed, considerations regarding data privacy and management would be necessary. Furthermore, these features entail agentic abilities beyond conversation, such as cross-checking participant and staff schedules and creating calendar appointments, which may involve greater potential for error than conversation alone. Some specific features such as consent verification or addressing questions regarding the consent form may require careful implementation and oversight, given the crucial ethical implications of informed consent. These options generated the highest rates of “not acceptable” responses, particularly among respondents conducting research in the “more than minimal risk” category.

This study identified potential features and offered preliminary observations regarding the development of a prototype. Limitations include the small, single institution sample, which may reduce the generalizability of findings, and the administration of a follow-up survey to all participants, that is, those who attended focus groups and those who did not, as responses from attendees may be biased by having attended a focus group. More formative work may be required to further validate and refine these findings, including engaging diverse potential participant populations and conducting iterative prototype development and usability assessments.

## Appendix

Multimedia Appendix 1 Pre–focus group survey.

Multimedia Appendix 2 Semistructured questions.

Multimedia Appendix 3 Follow-up survey.

## Abbreviations

AI-based VSA: artificial intelligence–based virtual study assistant
GenAI: generative artificial intelligence
UW: University of Washington
