# Supplementary material for: Exploring Feature Priorities and User Needs in Developing Virtual Study Assistants
Source: JMIR Form Res. 2026 Mar 6;10:e86945. doi: 10.2196/86945 (PMC13005061; doi:10.2196/86945)
Supplement: Multimedia Appendix 1 [file formative_v10i1e86945_app1.pdf]

Appendix 1. Prefocus group survey

Demographic Background

1. What is your age?

☐ 20–24 ☐ 25–29 ☐ 30–34 ☐ 35–39 ☐ 40–44 ☐ 45–49 ☐ 50–54

☐ 55–59 ☐ 60–64 ☐ 65–69 ☐ ≥ 70

2. What broad racial group do you identify with? Select all that apply.

☐ American Indian or Alaska Native ☐ Asian ☐ Black or African American

☐ Native Hawaiian or Other Pacific Islander ☐ White

☐ Other: __________ ☐ Prefer not to answer

3. Ethnicity

☐ Hispanic or Latino ☐ Not Hispanic or Latino ☐ Prefer not to answer

4. **Please choose your gender/gender identity:**

☐ Woman ☐ Man ☐ Transgender / Trans Woman ☐ Transgender / Trans Man

☐ Non-Binary ☐ Not listed: __________ ☐ Prefer not to answer

Prefocus Group Survey

1. Over your whole work career, about how long have you worked in human subjects research?

☐ Up to 1 year ☐ 1–2 years ☐ 2–3 years ☐ 3–4 years ☐ 4–5 years

☐ 5–6 years ☐ 6–7 years ☐ 7–8 years ☐ 8–9 years ☐ 9–10 years

☐ More than 10 years

2. What is your current job title?

☐ Research scientist / principal investigator ☐ Grants administrator

☐ Interventionist ☐ Research coordinator / assistant / consultant

☐ Other: __________

3. Which of these options best describes your education?

☐ High school degree

☐ Associate's degree or other two-year college degree

☐ Bachelor's degree or other four-year college degree

☐ Master's degree

☐ Academic doctoral degree

☐ Professional doctoral degree (examples: MD, JD, DPT, DNP, PsyD)

4. What types of human subjects studies have you worked on? Select all that apply.

☐ Interventional studies, such as clinical trials

☐ Dissemination and implementation trials

☐ Observational studies with biospecimens or behavioral testing

☐ Observational studies with surveys only

☐ Qualitative studies

☐ Secondary data analyses, EMR studies, or similar

☐ Other: __________

5. Have you ever worked on a study where at least some participants were recruited, screened, and enrolled entirely remotely?

☐ Yes ☐ No

If Yes:

5.i. What kinds of studies have you worked on where recruitment, screening, and enrollment were done entirely remotely for at least some participants? Select all that apply.

☐ Interventional studies, such as clinical trials

☐ Dissemination and implementation studies

☐ Observational studies with biospecimens or behavioral testing

☐ Observational studies with surveys only

☐ Qualitative studies ☐ Other: __________

6. Have you ever worked on a study where all study procedures from recruitment to data collection were conducted entirely remotely?

☐ Yes ☐ No

If Yes:

6.i. What kinds of studies have you worked on where all study procedures were conducted entirely remotely? Select all that apply.

☐ Interventional studies, such as clinical trials

☐ Dissemination and implementation studies

☐ Observational studies with biospecimens or behavioral testing

☐ Observational studies with surveys only

☐ Qualitative studies

☐ Other: __________

7. Have you ever heard of generative artificial intelligence (AI), also called "gen AI"?

☐ Yes ☐ No

8. Have you ever heard of any of these gen AI chatbots? Select all that apply.

☐ ChatGPT ☐ Copilot ☐ Gemini ☐ Claude ☐ Llama

☐ Other: __________ ☐ None of above

9.i. Which chatbots have you used or interacted with for personal use? Select all that apply.

☐ ChatGPT ☐ Copilot ☐ Gemini ☐ Claude ☐ Llama

☐ Other: __________ ☐ None of above

9.ii. Which chatbots have you used or interacted with for professional use, that is as part of your job? Select all that apply.

☐ ChatGPT ☐ Copilot ☐ Gemini ☐ Claude ☐ Llama

☐ Other: __________ ☐ None of above
