# Supplementary material for: Exploring Feature Priorities and User Needs in Developing Virtual Study Assistants
Source: JMIR Form Res. 2026 Mar 6;10:e86945. doi: 10.2196/86945 (PMC13005061; doi:10.2196/86945)
Supplement: Multimedia Appendix 2 [file formative_v10i1e86945_app2.pdf]

Appendix 2. Semistructured questions

PERCEPTION OF VSA:

1. What are your initial thoughts on using a VSA in research studies?
2. What aspects of your work could lend themselves to a VSA?
3. What key features do you think a VSA should have to effectively support the tasks?
4. Compared to humans, in what areas do you think the VSA could potentially perform better, and in what areas might it perform worse?
5. What aspects of your work would need to be maintained by a human?
6. Do you have any concerns about using a VSA tool in research studies?
7. Could you elaborate on any specific concerns?
