# Supplementary material for: Exploring Feature Priorities and User Needs in Developing Virtual Study Assistants
Source: JMIR Form Res. 2026 Mar 6;10:e86945. doi: 10.2196/86945 (PMC13005061; doi:10.2196/86945)
Supplement: Multimedia Appendix 3 [file formative_v10i1e86945_app3.pdf]

**Appendix 3. Follow up survey**

1. What risk category (or categories) of human subjects studies have you been involved in? Select all that apply.
   ☐ More than minimal risk (full IRB review) ☐ No more than minimal risk (expedited review)

   ☐ Exempt

**Feature 1: A virtual study chatbot translating study documents into other languages**

How acceptable would this be to you?

☐ Not acceptable ☐ Somewhat acceptable ☐ Moderately acceptable

☐ Very acceptable ☐ Extremely acceptable ☐ No opinion

**Feature 2: A virtual study chatbot reaching out to ask if a potential participant is interested in the study**

How acceptable would this be to you?

☐ Not acceptable ☐ Somewhat acceptable ☐ Moderately acceptable

☐ Very acceptable ☐ Extremely acceptable ☐ No opinion

**Feature 3: A virtual study chatbot asking and answering questions about eligibility for the study**

How acceptable would this be to you?

☐ Not acceptable ☐ Somewhat acceptable ☐ Moderately acceptable

☐ Very acceptable ☐ Extremely acceptable ☐ No opinion

**Feature 4: A virtual study chatbot scheduling participant interactions**

How acceptable would this be to you?

☐ Not acceptable ☐ Somewhat acceptable ☐ Moderately acceptable

☐ Very acceptable ☐ Extremely acceptable ☐ No opinion

**Feature 5: A virtual study chatbot describing the study to participants**

How acceptable would this be to you?

☐ Not acceptable ☐ Somewhat acceptable ☐ Moderately acceptable

☐ Very acceptable ☐ Extremely acceptable ☐ No opinion

**Feature 6: A virtual study chatbot answering questions from participants about the consent form and/or participation in the study**

How acceptable would this be to you?

☐ Not acceptable ☐ Somewhat acceptable ☐ Moderately acceptable

☐ Very acceptable ☐ Extremely acceptable ☐ No opinion

**Feature 7: A virtual study chatbot verifying participant understanding of consent**

How acceptable would this be to you?

☐ Not acceptable ☐ Somewhat acceptable ☐ Moderately acceptable

☐ Very acceptable ☐ Extremely acceptable ☐ No opinion

**Feature 8: A virtual study chatbot verifying eligibility**

How acceptable would this be to you?

☐ Not acceptable ☐ Somewhat acceptable ☐ Moderately acceptable
☐ Very acceptable ☐ Extremely acceptable ☐ No opinion

***Now consider which of these features you would most want in a virtual study chatbot. Please rank these features in order of highest (1) to lowest priority (8).***

| | 1 | 2 | 3 | 4 | 5 | 6 | 7 | 8 |
|---|---|---|---|---|---|---|---|---|
| A virtual study chatbot transcribing recorded interviews or focus groups | ☐ | ☐ | ☐ | ☐ | ☐ | ☐ | ☐ | ☐ |
| A virtual study chatbot reaching out to ask if a potential participant is interested in the study | ☐ | ☐ | ☐ | ☐ | ☐ | ☐ | ☐ | ☐ |
| A virtual study chatbot asking and answering questions about eligibility for the study | ☐ | ☐ | ☐ | ☐ | ☐ | ☐ | ☐ | ☐ |
| A virtual study chatbot scheduling participant interactions | ☐ | ☐ | ☐ | ☐ | ☐ | ☐ | ☐ | ☐ |
| A virtual study chatbot describing the study to participants | ☐ | ☐ | ☐ | ☐ | ☐ | ☐ | ☐ | ☐ |
| A virtual study chatbot answering questions from participants about the consent form and/or participation in the study | ☐ | ☐ | ☐ | ☐ | ☐ | ☐ | ☐ | ☐ |
| A virtual study chatbot verifying participant understanding of consent | ☐ | ☐ | ☐ | ☐ | ☐ | ☐ | ☐ | ☐ |
| A virtual study chatbot verifying eligibility | ☐ | ☐ | ☐ | ☐ | ☐ | ☐ | ☐ | ☐ |
